# Psoralen Isolated from the Roots of *Dorstenia psilurus* Welw. Modulate Th1/Th2 Cytokines and Inflammatory Enzymes in LPS-Stimulated RAW 264.7 Macrophages

**DOI:** 10.1155/2024/8233689

**Published:** 2024-07-11

**Authors:** Adamu Imam Isa, Hugues Fouotsa, Osama A. Mohammed, Mushabab Alghamdi, Bappa Adamu, Jaber Alfaifi, Abubakar Mohammed Jibo, Mohannad Mohammed Saleh Alamri, Sameer Khan, Masoud Ishag Elkhalifa Adam, Abdullah Ali Alqarni, Mohamed O'haj Mohamed, Joël Eddy Terence Ateba, Jean Paul Dzoyem

**Affiliations:** ^1^ Department of Physiology College of Medicine University of Bisha, Bisha 61922, Saudi Arabia; ^2^ Department of Process Engineering National Higher Polytechnic School of Douala University of Douala, Douala, Cameroon; ^3^ Department of Pharmacology College of Medicine University of Bisha, Bisha 61922, Saudi Arabia; ^4^ Department of Internal Medicine College of Medicine University of Bisha, P.O. Box 3752, Bisha, Asir 67713, Saudi Arabia; ^5^ Department of Child Health College of Medicine University of Bisha, Bisha 61922, Saudi Arabia; ^6^ Department of Family and Community Medicine College of Medicine University of Bisha, Bisha 61922, Saudi Arabia; ^7^ Department of Medical Education and Department of Medicine College of Medicine University of Bisha, Bisha, Saudi Arabia; ^8^ Department of Clinical Biochemistry College of Medicine University of Bisha, Bisha 61922, Saudi Arabia; ^9^ Department of Biochemistry Faculty of Science University of Dschang, Dschang, Cameroon

## Abstract

*Dorstenia psilurus* is a widely used plant spice in traditional African medicine to treat pain-related conditions. However, the anti-inflammatory mechanisms underlying this activity and the main active ingredients of *D. psilurus* have not yet been fully characterized. This study aimed to isolate and identify the main active anti-inflammatory constituents of the *D. psilurus* extract and to investigate the underlying anti-inflammatory mechanisms in murine macrophages. Chromatographic techniques and spectroscopic data were used for compound isolation and structure elucidation. The Griess reagent method and the ferrous oxidation-xylenol orange assay were used to evaluate the inhibition of NO production and 15-lipoxygenase activity, respectively. Cyclooxygenase activity was assessed using the fluorometric COX activity assay kit, and Th1/Th2 cytokine measurement was performed using a flow cytometer. The results indicated that the extract and fractions of *D. psilurus* inhibit NO production and proliferation of RAW 264.7 macrophage cells. Bioguided fractionation led to the identification of psoralen, a furocoumarin, as the main bioactive anti-inflammatory compound. Psoralen inhibited NO production and 15-lipoxygenase activity and reduced pro-inflammatory Th1 cytokines (IFN-*γ*, TNF-*α*, and IL-2) while increasing the secretion of anti-inflammatory cytokines (IL-4, IL-6, and IL-10) in activated RAW 264.7 macrophage cells. The encouraging results obtained in this study suggest that psoralen-based multiple modulation strategies could be a useful approach to address the treatment of inflammatory diseases.

## 1. Introduction

Inflammation, a physiological response to injury or infection, represents a double-edged sword in the human body [1]. Although acute inflammation is a crucial defense mechanism, chronic inflammation is the basis of several debilitating diseases, from rheumatoid arthritis to cardiovascular disorders [2]. The limitations and side effects associated with conventional anti-inflammatory drugs have led to the exploration of natural sources. Medicinal plants, with their large diversity of bioactive chemicals, offer a repertoire of bioactive compounds that can serve as a source for novel drug development [3]. In this context, the *Dorstenia* species has garnered attention due to its role in the discovery of potential compounds for the treatment of inflammatory diseases [4].

The genus *Dorstenia* Linne (Moraceae) is globally represented by approximately 170 species [5]. It predominantly consists of undergrowth and herbaceous perennials with succulent and scrambling rhizomes [6]. The initial documentation of a plant of this genus was presented by Casagrande et al. [7] in 1974, where they observed the presence of steroids. In 1988, *Dorstenia barnimiana* from Ethiopia was reported to contain unusual styrenes and benzofuran derivatives [8]. Interest in this genus has been steadily growing during the past decade, resulting in the publication of nearly 40 papers on more than 25 species of *Dorstenia*. Many *Dorstenia* species possess significant medicinal properties and are utilized in culturally established medical practices in numerous countries. They are used as anti-snakebite, anti-infection, and antirheumatic remedies in the medicinal plant therapy of several African and Central and South American countries [9]. *D. psilurus* in Cameroon, *D. brasiliensis* in Brazil, and *D. contrajerva* in Panama and Mexico are specifically employed for these purposes. In Addis Ababa, Ethiopia, *D. barnimiana* (known locally as Worq bemeda) is of great importance as a medicinal plant, its tubers being sold in Merkato for the treatment of various diseases, most notably gout [10]. In Cameroon, these plants are commonly used for the treatment of infections and wounds [11]. *D. elleptica* is particularly utilized for the treatment of eye infections. In the cultural medicine of Brazil, a drug formulation called “*Carapia*” is based on *Dorstenia* species and is used for the treatment of skin diseases [9]. In Cameroon, a decoction of *D. psilurus* is used to treat rheumatism, snake bites, headache, and stomach disorders [9]. The widespread use of *Dorstenia* species in traditional medicine around the world has led to extensive research on this genus [12]. During the past two decades, there has been a surge in research on the *Dorstenia* genus, with numerous studies focusing on the chemistry of various species [12].

In addition to conventional sterols and triterpenes, the genus represents a large reservoir of furocoumarin (turbinatocoumarin from the twigs of *D. turbinate* [13]), styrenes, benzofuran derivatives (dorsjervin A and dorsjervin B from *D. contrajerva* [14], dorstenin and bergapten from *D. elliptica* [15]), and C-prenylated flavonoids (dorsilurin [16]). Benzofuran and coumarin derivatives have been isolated from nine different species of *Dorstenia*, the majority of which originate from Central and South America. These species include *D. brasiliensis* from Brazil [17], *D. excentrica*, *D. drakena*, and *D. lindeniana* from Mexico [18], and *D. contrajerva* from Panama [19] and Mexico [20]. The remaining species, *D. barnimiana*, is found in Ethiopia [8]. Additionally, *D. poinsettifolia* and *D. psilurus* are native to Cameroon [21]. Previous phytochemical analysis of *D. psilurus* revealed a large amount of triprenylated flavonols, including dorsilurin F and G [16].

The complex interaction between some bioactive natural products and inflammatory pathways is noteworthy for investigation, seeking not only to elucidate their impact on key enzymes and mediators but also to understand their influence on the delicate balance of Th1/Th2 cytokines in macrophages. Nitric oxide synthase (NOS) is an enzyme responsible for catalyzing the production of nitric oxide (NO) from the amino acid L-arginine [22]. There are three isoforms of NOS: neuronal NOS (nNOS or NOS1), inducible NOS (iNOS or NOS2), and endothelial NOS (eNOS or NOS3) [22]. Inducible NOS (iNOS) is of particular interest due to its role in the immune response and inflammation. Its expression can be induced by a wide range of stimuli, such as lipopolysaccharides (LPS) and bacterial or viral infections, leading to sustained production of high levels of NO [23]. iNOS-mediated NO production is involved in various pathological conditions, including septic shock and chronic inflammatory disorders. Its regulation and potential therapeutic targeting remain areas of active research in the fields of immunology, inflammation, and drug development [23]. Nitric oxide inhibitors are promising therapeutic agents for treating various immune and inflammatory conditions [24]. At the molecular level, the inflammatory enzymes cyclooxygenase (COX) and lipoxygenase (LOX) play a pivotal role in the initiation and progression of inflammation [25]. COX, which exists in two isoforms (COX-1 and COX-2), catalyzes the conversion of arachidonic acid to prostaglandins, while LOX is responsible for the synthesis of leukotrienes [26, 27]. Both pathways are central players in the amplification of inflammatory signals. The ability of natural compounds to modulate these enzymes represents a potential avenue for the development of novel anti-inflammatory therapies. In addition to their impact on inflammatory enzymes, several bioactive natural products exhibit intriguing immune-modulatory properties [28]. The intricate network of Th1 and Th2 cytokines, monitored by T-helper cells, orchestrates the immune response. Imbalances in this network are implicated in various inflammatory disorders [29]. By investigating the influence of *D. psilurus* bioactive compounds on Th1/Th2 cytokine balance in RAW 264.7 macrophages stimulated by LPS, our objective is to decipher their role in shaping the immune environment during inflammation. As a component of our ongoing investigation into natural substances as potential sources of new pharmaceutical agents targeting pain and inflammatory conditions, psoralen (1), octadecanyl-3 [4-hydroxyphenyl] -prop-2-enoate (2), and sitosterol glucoside (3) were isolated from a methanol extract of roots of *D. psilurus* to assess their impact in vitro on nitric oxide (NO) production, inhibition of COX-1/COX-2/15-LOX, and production of Th1/Th2 cytokines in activated macrophages RAW 264.7 activated macrophages.

## 2. Materials and Methods

### 2.1. General Procedures

The ^1^H, ^13^C, and 2D NMR spectra were obtained using Bruker ARX, specifically the ^1^H 400 and ^13^C 100 MHz, ^1^H 500 and ^13^C 125 MHz, and ^1^H 600 and ^13^C 150 MHz spectrometers, all provided by Bruker BioSpin GmbH in Rheinstetten, Germany. CD_3_OD and CDCl_3_ were used as solvents for these NMR experiments. The COSY 458 experiment was used to determine homonuclear ^1^H─^1^H connectivities. One-bond ^1^H─^13^C connectivities were determined through HMQC, while two and three-bond ^1^H─^13^C connectivities were determined through HMBC experiments. The chemical changes of the proton were reported in *δ* (ppm) relative to the residual signal of CDCl_3_ signal at d 7.26, and ^13^C NMR spectra were referenced to the central peak of CDCl_3_ at 77.0. Coupling constants (*J*) were measured in Hz. *m.p* values; IR, UV, and ESI-MS data were collected from the literature to complete the obtained NMR data. Column chromatography (CC) was performed using silica gel 60 (70–230 and 240–300 mesh, E. Merck) and Sephadex LH-20 (GE Healthcare Bio-Sciences AB, Uppsala, Sweden). Preparative thin-layer chromatography (PTLC) was performed on F254 PTLC plates provided by E. Merck in Darmstadt, Germany. Precoated silica gel TLC was used to assess the purity of the compound, while ceric sulfate spray reagent was used to visualize compounds on TLC plates.

### 2.2. Plant Material

The roots and the entire *Dorstenia psilurus* Welwistsch plant were harvested from Mbouda (5.6235° N, 10.2544° E) in the western region of Cameroon in August 2018. The entire plant was identified at the Cameroon National Herbarium (HNC) in Yaounde under the voucher code 44839/HNC and the plant name *D. psilurus* Welw. (Moraceae) was authenticated using the plant index database, World Flora Online, under the reference code WFO-0000654605.

### 2.3. Extraction and Isolation

The exhaustive extraction of methanol (5 L) was performed three times for 3 days at room temperature on the ground and air-dried roots of *D. psilurus* (700 g). The resulting extract, after concentration under reduced pressure, produced a crude extract (DP, 65.0 g). Subsequently, this crude extract was partitioned into petroleum ether (20.0 g), dichloromethane (13.0 g), ethyl acetate (17.0 g), and methanol residue (15.0 g), resulting in the production of four dark brown extracts known as DPA, DPB, DPC, and DPD extracts, respectively. The CC was then used on the petroleum ether extract (DPA, 20.0 g) using a silica gel column (70–230 mesh; 200.0 g). The elution was carried out using 100% petroleum ether, followed by a petroleum ether-EtOAc gradient, increasing in polarity from PE-EtOAc (95 : 5) to EtOAc 100%. This process resulted in the collection of 74 subfractions, each measuring 100 mL. The subfractions were classified as petroleum ether 100% (subfractions 1–15), petroleum ether-EtOAc 90 : 10 (16–25), petroleum ether-EtOAc 80 : 20 (26–34), petroleum ether-EtOAc 65 : 35 (35–47), petroleum ether-EtOAc 50 : 50 (48–53), petroleum ether-EtOAc 25 : 75 (54–65) and EtOAc 0 : 100 (66–74). Based on their analytical thin layer chromatography (TLC) profile, these fractions were combined into seven distinct fractions, namely DPA1 (subfractions 1–10; 2.4 g), DPA2 (subfractions 11–20; 3.7 g), DPA3 (subfractions 21–33; 5.3 g), DPA4 (subfractions 34–47; 2 g), DPA5 (subfractions 48–59; 2.54 g), DPA6 (subfractions 60–71; 1.72 g) and DPA7 (subfractions 72–74; 1.2 g). The DPA2 fraction was subjected to rechromatography, resulting in the isolation of octadecanyl-4-hydroxycinnamate (2; 80 mg). Further purification of the fraction DPA3, through CC and recrystallization, led to the acquisition of psoralen (1; 25 mg).

For the DPC of EtOAc extract (17.0 g), chromatography was performed on a silica gel 60 (100 g) column, starting with 100% dichloromethane (DCM) and gradually transitioning to a mixture of DCM-MeOH (97 : 3), until finally reaching MeOH 100%. As a result, 36 subfractions were obtained and subsequently evaporated using a rotary evaporator. Based on TLC, the subfractions were combined into five distinct fractions: DPC1 (subfractions 1–8; 1.45 g), DPC2 (subfractions 9–17; 2.0 g), DPC3 (subfractions 18–25; 4.2 g), DPC4 (subfractions 26–31; 3.24 g), and DPC5 (subfractions 32–36; 1.87 g). The DPC3 fraction (subfractions 18–25; 4.2 g) was further purified by CC and, in some cases, with the help of successive preparative TLC, resulting in the isolation of *β*-sitosterol glucoside (3; 35 mg). All the compounds obtained in this study were previously known and, therefore, were identified by means of ^1^H and ^13^C NMR analysis.

*Psoralen* (1): White needles; mp. 163°C. ESI–MS *m/z* (rel. int.): 186 (100). UV *λ*_max_ (MeOH) nm (log *ε*): 240 (4.18), 290 (3.80), 320 (3.50). IR *ʋ*_max_ cm^−1^ 1,700, 1,620, 1,580, 1,500. ^1^H NMR (500 MHz, Methanol-*d*_4_): *δ* 6.34 (1H; d; *J* = 9.5 Hz; H-3), 7.94 (1H; d; *J* = 9.5 Hz; H-4), 7.46 (1H; brs; H-8), 7.75 (1H; d; *J* = 2.3; H-2′), 6.88 (1H; dd; *J* = 2.3; 1.0 Hz; H-3′), 7.77 (1H; brs; H-5). ^13^C NMR (125 MHz, methanol-*d*_4_): *δ* 162.8 (C-2), 157.2 (C-7), 152.5 (C-8a), 147.9 (C-2′), 145.9 (C-4), 127.1 (C-6), 120.9 (C-5). 115.1 (C-4a), 114.5 (C-3), 107.0 (C-3′). 100.1 (C-8) [21] (Supplementary materials S1, S2, S3, S4, S5, and S6).

*Stearyl ferulate* (*Octadecanyl-3*[*4-hydroxyphenyl*]*-prop-2-enoate*) (2): White powder, m.p. 97−99°C. ESI–MS *m/z* (rel int): 446 [*M* + 1]^+^. UV *λ*_max_ (MeOH) nm (log *ε*): 203.5 (3.97). 228.0 (3.85), 311.5 (4.12), 424.5 (2.68). ^1^H NMR (400 MHz, Acetone-*d*_6_): *δ* 0.82 (3H; t; *J* = 6.8 Hz; CH_3_), 4.15 (2H; t; *J* = 7.0 Hz; OCH_2_), 6.23 (1H; d; *J* = 15.9 Hz; H-*α*), 6.78 (2H; brd*; J =* 8.6 Hz; H-3; H-5). 7.38 (2H; brd; *J* = 8.6 Hz; H-2; H-6), 7.41 (1H; brs; -OH), 7.56 (1H; d*; J =* 15.9 H; H-*β*). ^13^C NMR (100 MHz, Acetone-*d*_6_): *δ* 167.9 (C-9), 145.8, 144.8 (C-3, C-4), 144.6 (C-7), 128.0 (C-1), 123.1 (C-6), 116.8 (C-8), 114.7 (C-5), 109.3 (C-2), 64.7 (C-l'), 55.0 (-OMe), 31.0 (C-2′), 29.8 (C-3′), 29.4 (C-4′-16′), 14.2 (Me) [30] (Supplementary materials S7, S8, S9, S10, and S11).

*β-sitosterol glucoside* (3): White microcrystalline powder, m.p. 290°C. ESI–MS *m*/*z* (rel int): 576 [*M* + 1]^+^. C_35_H_60_O_6_. IR *ʋ*_max_ (cm^−1^): (3,600–3,400), 2,900, 2,850, 1,720, 1,640, 1,450, 1,240, 900, (830–800) cm^−1^; ^1^H-NMR (600 Mz, DMSO-*d*6): 1.00–1.40 (2H; m; H-1), 1.58–1.26 (2H; m; H-2), 2.98 (1H; m; H-3), 2.26 (1H; dt; *J* = 4.69; 8.10; H-4)-1.98 (1H; ddd; *J* = 1.98;12.94; 12.94; H-4), 5.35 (1H; t; *J* = 3.6; H-6), 1.73 (1H; ddd; *J* = 2.5, 7.0, 16.0; H-7) −1.95 (1H; ddd, *J* = 16.0, 2.5, 7.0; H-7), 1.36 (1H; m; H-8), 0.85 (1H; m; H-9), 1.42 (2H; m; H-11), 1.52 (1H; dd; *J =* 4.3;12.37; H-12) −1.20 (1H; m; H-12), 0.95 (1H; m; H-14), 1.05 (1H; m; H-15)−1.57 (1 H; m; H−15), 1.25 (1H; m; H-16)−1.85 (1H; m; H−16), 1.20 (1H; m; H-17), 0.70 (3H; 5; H-18), 0.94 (3H; 5; H−19), 1.40 (1H; m; H−20), 0.95 (3 H; d; *J* = 6.5; H-21), 1.20 (2H; m; H−22), 1.25 (2 H; m; H-23), 0.94 (1H; m; H−24), 1.68 (1H; m; H-25), 0.87 (3H; d; *J* = 7.0; H-26), 0.88 (3H; d; *J* = 7.0; H−27), 1.30 (2H; m; H-28), 0.77 (3H; d; *J =* 7.5; H-29), 4.20 (1H; d; *J =* 7.9; H-1′), 2.89 (1H; dt; *J =* 4.5, 8.0; H-2′), 3.27 (1H; dt; *J =* 4.5; 8.0; H-3′), 3.00 (1H; dt; *J =* 4.5; 8.0; H-4′), 3.06 (1H; dt; *J =* 4.5; 8.0; H5′); 4.55 (1 hr; dd; *J =* 2.5; 11.77; H-6′) - 4.40 (1H; dd; *J =* 5.2; 11.77; H-6′). ^13^C-NMR (150 Mz, DMSO-*d*6): 36.9 (C-1), 29.3 (C-2), 76.9(C-3), 40.1 (C-4), 140.5 (C-5), 121.3 (C-6), 31.5 (C-7), 31.4 (C-8), 49.6 (C-9), 36.3 (C-10), 20.7 (C-11), 38.3 (C-12), 41.9 (C-13), 56.2 (C-14), 23.9 (C-15), 27.9 (C-16), 55.5 (C-17), 11.7 (C-18), 19.2 (C-19), 35.5 (C-20), 18.7 (C-21), 33.4 (C-22), 25.4 (C-23), 45.2 (C-24), 28.7 (C-25), 19.8 (C-26), 19.0 (C-27), 22.6 (C-28), 11.8 (C-29) and the chemical shifts 100.7 (C-1′), 73.5 (C-2′, 76.9 (C-3′), 70.1 (C-4′), 76.8 (C-5′), and 611 (C-6′) are due to the carbon of the sugar moiety [31] (Supplementary materials S12, S13, S14, S15, and S16).

### 2.4. Chemicals

Tris (hydroxymethyl) aminomethane was acquired from Sigma, located in Switzerland, while 15-lipoxygenase derived from *Glycine max*, ferrous sulfate, indomethacin, and sodium nitrite were obtained from Sigma, based in Germany. Penicillin/streptomycin/fungizone (PSF), Dulbecco's modified Eagle's medium (DMEM), and fetal calf serum (FCS) were purchased from Highveld Biological Products, situated in South Africa. The lysophosphate (LPS) sourced from *Escherichia coli* 0111: B4 was obtained from Sigma–Aldrich, located in Darmstadt, Germany. Whitehead Scientific, located in South Africa, provided trypsin and phosphate-buffered saline (PBS). 3-(4, 5-dimethylthiazol-2-yl)-2, 5-diphenyl-tetrazolium bromide (MTT) and quercetin were provided by Sigma–Aldrich, based in St. Louis, MO, USA. Linoleic acid was purchased from Merck, located in Darmstadt, xylenol orange from Searle, based in England, and sodium carbonate was purchased from Holpro Analytic, located in South Africa.

### 2.5. Nitric Oxide Inhibitory Activity in LPS-Activated RAW 264.7 Macrophages

#### 2.5.1. Cell Culture and Maintenance

The mouse-derived macrophage cell line RAW 264.7, purchased from the American Type Culture Collection (ATCC TIB-71, Rockville, MD, USA), was cultured in DMEM medium supplemented with 10% FCS and 1% penicillin/streptomycin/fungizone (PSF) under conventional cell culture conditions at a temperature of 37°C and a carbon dioxide concentration of 5% in a controlled humid atmosphere. Routine maintenance in culture (passage) was performed following ATCC instructions.

#### 2.5.2. Inhibition of Nitric Oxide (NO) Production in LPS-Activated RAW 264.7 Macrophages

The RAW 264.7 macrophage cells were introduced into 96 well microtiter plates at a density of 2 × 10^5^ cells/mL. Subsequently, cells were subjected to activation through incubation in a medium consisting of 1 *µ*g/mL LPS alone, acting as the control. Furthermore, lipopolysaccharide was combined with varying concentrations of samples dissolved in DMSO (concentration ranges were 200–1.56 *µ*g/mL for extracts and fractions and 100–0.78 *µ*M for compounds). Indomethacin, a well-known nonsteroidal anti-inflammatory drug, was used as a standard inhibitor of NO production [32]. The quantity of nitric oxide released from macrophages was assessed using Griess reagent as previously described [33]. All experiments for the measurement of nitric oxide inhibition were conducted three times in triplicate.

#### 2.5.3. Cytotoxicity Assay

To confirm that the observed inhibition of nitric oxide was not a result of cytotoxic effects, a cytotoxicity assay was performed, following the culture protocol established by Mosmann [34], with some minor adjustments. Subsequent to the removal of the media, cells were replenished with 200 ml of DMEM. After removal of the medium, the cells were topped with 200 mL of DMEM. In each well, 30 mL of 15 mg/mL of 3-(4, 5-dimethylthiazol-2-yl)-2, 5-diphenyl tetrazolium bromide (MTT) was added. Cells were incubated at 37°C with 5% CO_2_. After 2 hr, the medium was carefully discarded, and the formed formazan salt was dissolved in DMSO. The absorbance was read at 570 nm (SpectraMax 190, Molecular devices). The percentage of cell viability was calculated with reference to the control (untreated cells containing LPS taken as 100% viability). Doxorubicin was used as a standard cytotoxic drug [35].

### 2.6. Soybean LOX Inhibition Assay

The assay was conducted following a previously described methodology [36, 37] with minor adjustments. The assay is based on the measurement of the formation of the Fe3+/xylenol orange complex on a spectrophotometer at a wavelength of 560 nm. 15-LOX from *G. max* was incubated with samples or standard inhibitors at a temperature of 25°C for 5 min. The concentration ranges of the samples ranged from 200 to 1.56 *µ*g/mL for extracts and fractions and from 100 to 0.78 *µ*M for compounds. Subsequently, linoleic acid (at a final concentration of 140 *µ*M) in Tris–HCl buffer (at a pH of 7.4 and a concentration of 50 mM), and the mixture was incubated at a temperature of 25°C for 20 min in the absence of light. The assay was concluded by adding 100 *µ*L of FOX reagent, which consisted of sulfuric acid (at a concentration of 30 mM), xylenol orange (at a concentration of 100 *µ*M), and iron (II) sulfate (at a concentration of 100 *µ*M) in a methanol/water mixture (at a ratio of 9 : 1). For control, only the LOX solution and buffer were pipetted into the wells. The blanks (background) contained the LOX enzyme during incubation, but the substrate (linoleic acid) was added after the addition of the FOX reagent. Quercetin was used as a reference compound to inhibit the inhibition of 15-LOX activity [38]. The inhibitory activity of LOX was evaluated by determining the percentage of inhibition of hydroperoxide production based on changes in absorbance values at a wavelength of 560 nm after 30 min at a temperature of 25°C. The percentage inhibition was calculated using the following formula: % inhibition = ((*A*_control_–*A*_blank_)−(*A*_sample_−*A*_blank_)/(*A*_control_−*A*_blank_)) ×100. Here, *A*_control_ represents the absorbance of the control well, *A*_blank_ represents the absorbance of the blank well, and *A*_sample_ represents the absorbance of the sample well. The IC_50_ values of the samples demonstrating more than 50% inhibition were determined by plotting the percentage inhibition against the concentration of the extract.

### 2.7. Evaluation of COX-1 and COX-2 Activity

Evaluation of COX-1 and COX-2 enzyme activity was performed using the fluorometric COX activity assay kit provided by Biovision. RAW 264.7 cells were cultured at a density of 2 × 10^5^ cells/mL in a 48-well microplate and allowed to adhere for a period of 24 hr. Subsequently, cells were treated with LPS at a concentration of 0.1 *µ*g/mL and psoralen at concentrations of 5, 20, and 50 *µ*M. The cyclooxygenase enzyme activity assay was performed after a 24 hr incubation period. To initiate the assay, cells were removed using TNE buffer containing Tris (40 mM), NaCl (150 mM), and EDTA (1 mM), followed by a washing step using 1x PBS. Cells were then resuspended in 1 mL of 1x PBS, transferred to a 1.5 mL tube, and subjected to centrifugation at 500x *g* for 3 min. The resulting pellet was resuspended in 0.5 mL of lysis buffer containing a protease inhibitor cocktail, followed by vortexing and incubation at 4°C for 5 min. Subsequently, the cell lysate was centrifuged at 12,000 x *g* for 3 min, and the supernatant was collected for the COX activity assay. The fluorometric cyclooxygenase activity assay kit provided by Biovision was utilized according to the manufacturer's instructions to assess COX activity. This assay incorporates specific inhibitors for COX-1 and COX-2 enzymes, allowing differentiation of their respective activities and those of other peroxidases. A standard drug, indomethacin, was used at a concentration of 10 *µ*M was employed, and appropriate controls were included in the experimental design.

### 2.8. Measurement of Th1/Th2 Cytokines

RAW 264.7 cells were cultured at a concentration of 2 × 10^5^ cells/mL in a 48-well microplate and allowed to adhere for 24 hr. Subsequently, they were subjected to lipopolysaccharide (LPS) treatment at a concentration of 0.1 *µ*g/mL, along with psoralen at concentrations of 5, 20, and 50 *µ*M. The control groups consisted of untreated cells and cells treated with indomethacin at a concentration of 10 *µ*M. Following an incubation period of 24 hr, the supernatant was collected, and the levels of pro-inflammatory Th1 cytokines (IFN-*γ*, TNF-*α*, and IL-2), as well as anti-inflammatory Th2 cytokines (IL-4, IL-6, and IL-10) were quantified. The experimental protocol adhered to the manufacturer's guidelines and utilized the BD TM cytometric beads array human Th1/Th2 cytokine kit (BD-Biosciences). Data acquisition was performed using a BD LSR FortessaTM cell analyzer flow cytometer.

### 2.9. Statistical Analysis

Data are displayed in the form of the average ± standard deviation (SD) of three separate trials or triplicate (*n* = 3). Disparities among the averages of each group were evaluated through a two-way analysis of variance, pursued by Dunnett's multiple comparison tests employing GraphPad Prism 9.

## 3. Results and Discussion

### 3.1. Bioguided Fractionation of *Dorsteni Psilurus* Extract

**Figure 1:**
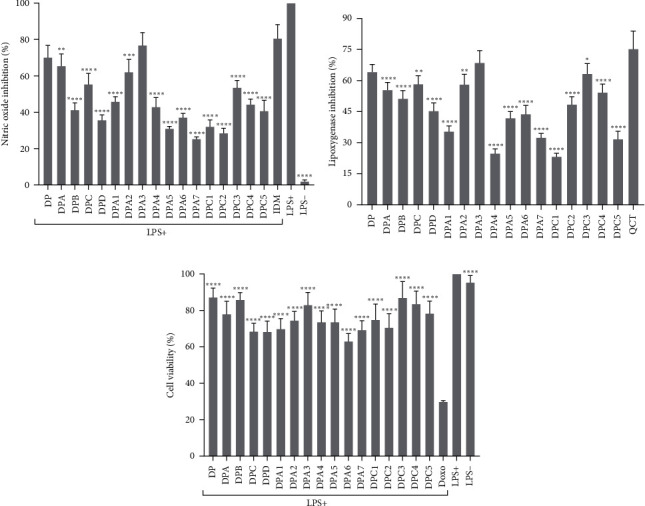
Percentage of inhibition of NO production (a), inhibition of 15-lipoxygenase activity (b), and viability of RAW 264.7 cells (c) of the crude extract, subextracts, and fractions of *D. psilurus* for the bioguided fractionation process. Statistical analysis was performed with Dunnett's multiple comparison test using two-way ANOVA.  ^*∗*^*p*  < 0.01,  ^*∗∗*^*p*  < 0.01,  ^*∗∗∗*^*p*  < 0.01,  ^*∗∗∗∗*^*p*  < 0.0001 between samples and reference drug. DP, crude extract; DPA, petroleum ether subextract; DPB, dichloromethane subextract; DPC, ethyl acetate subextract; DPD, methanol residue; DPA1–DPA7: fractions of DPA; DPC1–DPC5: DPC fractions. The samples were tested at a single concentration of 200 *μ*g/mL while doxorubicin and indomethacin were tested each at 10 *μ*M and quercetin at 50 *μ*M.

A bioguided fractionation assay was performed to separate the bioactive anti-inflammatory compounds that exist in the roots of *D. psilurus*. The crude methanol extract was divided into three subextracts using solvents with varying polarities, resulting in petroleum ether (DPA), dichloromethane (DPB), and ethyl acetate (DPC) subextracts. The different partitions obtained were tested for inhibition of nitric oxide and inhibition of 15-LOX activity at 200 *μ*g/mL. From the results presented in Figures 1(a) and 1(b), DPA and DPC subextracts were the most active both for NO production and 15-LOX inhibition and were then selected for fractionation. Then, the different fractions obtained were tested for inhibition of NO and 15-LOX at 200 *μ*g/mL. As shown in Figures 1(a) and 1(b), the percentage of inhibition varies between samples, ranging from 25.34% (DPA7) to 74.85% (DPA3) for NO production and from 23.21% (DPC1) to 68.58% (DPA3) for 15-LOX activity. In particular, high NO inhibitory activities were observed with DPA3 (74.85%) and DPA2 (62.06%). DPA3 had the highest 15-LOX inhibition (68.58%).

To determine whether NO inhibition was not due to the toxic effect of the samples, cell viability was performed. As illustrated in Figure 1(c), none of the subextracts tested appeared to be cytotoxic at the concentration tested. All samples showed more than 50% cell viability, with values ranging from 63.05% (DPA6) to 87.20% (DP).

Long-term use of nonsteroidal anti-inflammatory drugs (NSAIDs) such as ibuprofen, indomethacin, and diclofenac has been associated with damage to the gastrointestinal mucosa and an increased risk of cardiovascular disease [39]. Therefore, considerable attention has been focused on the discovery of new NSAIDS with improved biological activities and minimized toxicity from medicinal plants [40]. The comprehensive results of bioguided fractionation demonstrated that the unrefined extract and subextracts derived from *D. psilurus* exhibit anti-inflammatory characteristics through inhibition of NO production and 15-LOX activity. These findings support the customary application of *Dorstenia* species, as certain folkloric uses of the *Dorstenia* genus involve the alleviation of pain and inflammation [11]. *D. psilurus decomposition* is used in Cameroon as an analgesic and also to treat inflammatory and pain conditions such as rheumatism and headaches [41, 42]. From the crude extract to fractions, NO production and 15-LOX inhibitory activities increased with fractionation. These findings suggest that further studies, including dose-response analyses, isolation, and identification of active compounds from the most active fractions, were necessary to understand the underlying mechanisms and potential therapeutic applications. To this end, the DPA2, DPA3, and DPC3 fractions, which were the most active for both NO production and 15-LOX inhibition, were selected for compound isolation.

### 3.2. Isolation and Structural Elucidation of Compounds

**Figure 2:**
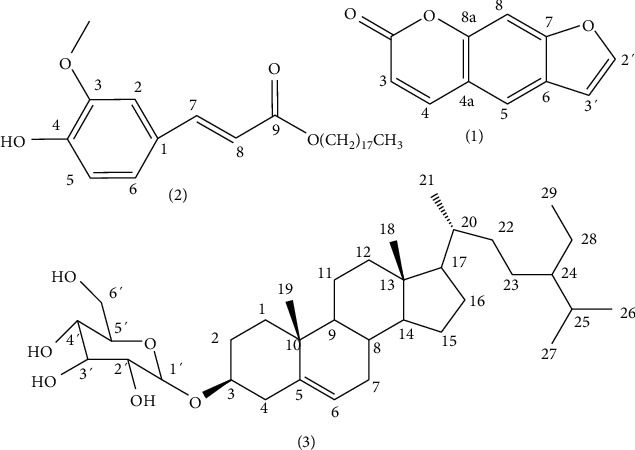
Chemical structures of isolated compounds of *Dorsteni psilurus* (1) psoralen, (2) octadecanyl-3[4-hydroxyphenyl]-prop-2-enoate (stearyl ferulate), and (3): *β*-sitosterol glucoside.

Chemical composition of individual substances obtained from the roots of *D. psilurus* Welw. (Moraceae) (Figure 2) were determined by analyzing their spectral data (^1^H, ^13^C, HSQC, and HMBC) and a comparative examination of these findings with previously published results. The aforementioned compounds have been recognized as psoralen. (1; white needles; C_11_H_6_O_3_; m.p.: 163°C; *m/z* 186; octadecanyl-3[4-hydroxyphenyl]-prop-2-enoate, (2; white powder; C_28_H_46_O_4_; m.p.: 97−99°C; *m/z* 446) and *β*-sitosterol glucoside, and (3; white microcrystalline powder; C_35_H_60_O_6_; m.p.: 290°C; *m/z* 576).

**Figure 3:**
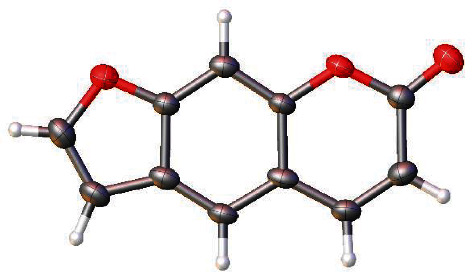
Ortep-like plot of psoralen in the crystal. Thermal parameters are shown at the 50% level.

*X-ray structure of psoralen*: The structure of 1 was first described by Bideau et al. [43]. Structural measurements were repeated at a temperature of 100 K to acquire more accurate and detailed information about the structure, as depicted in Figure 3. Supplementary crystallographic data can be found in CCDC 2011897. Interested individuals can access these data at no cost from the Cambridge Crystallographic Data Centre via the website https://www.ccdc.cam.ac.uk/conts/retrieving.html.

### 3.3. NO Production and Inhibition of LOX Activity of the Selected Actives Extract, Subextracts, Fractions, and Isolated Compounds

To confirm NO production and 15-LOX inhibitory activities of the most active extract, subextracts, and fraction, dose-response experiments were performed for samples with more than 50% inhibition. These experiments were also extended to isolated compounds. This allowed us to determine the IC_50_ values (Table 1). All of the examined samples exhibited activities that were dependent on the dose. Both for NO production and 15-LOX inhibition, DPA3 exhibited the lowest IC_50_ values (87.81 and 67.85 *μ*g/mL, respectively), confirming its high potential effects on inflammation. DPA3 appeared to be the most promising fraction, both for the inhibition of NO and 15-LOX. DP and DPA2 also exhibit moderate activities against both targets, emphasizing their potential to also contain some bioactive anti-inflammatory constituents.

Dose-dependent NO production and 15-LOX inhibitory activities of the isolated compounds were also carried out, and the IC_50_ values were determined (Table 1).

The results indicated that among the three isolated compounds, compound (1) was the most potent, with the lowest IC_50_ values for both NO inhibition (1.19 *μ*g/mL) and 15-LOX inhibition (3.43 *μ*g/mL). This result is in line with the literature. Psoralen is the main active ingredient found in the plant of the genus *Psoralea* [44]. Previous research has provided evidence that psoralen exhibits several bioactive characteristics, such as its potential in the treatment of osteoporosis and inflammation [45]. There have been reports on the inhibitory effects of certain derivatives of psoralen, such as xanthotoxol, on the production of prostaglandin E2 in RAW 264.7 cells stimulated by lipopolysaccharide [46]. However, this is the first study to focus on the ability of psoralen to interfere with NO production and 15-LOX activity. Compound (2), although less potent than compound (1), demonstrated moderate efficacy with IC_50_ values of 39.41 *μ*g/mL and 35.57 *μ*g/mL for NO production and 15-LOX, respectively. On the contrary, compound (3) exhibited a lower potency on NO production with IC_50_ values of 26.15 *μ*g/mL, suggesting a limited inhibitory effect compared to quercetin. However, in a study by Choi et al. [47], *β*-sitosterol-*β-D*-glucoside isolated from *Trachelospermum jasminoides* was found to reduce NO production by 72.8% in LPS-stimulated RAW 264.7 murine macrophages. Compound (3) demonstrated notable 15-LOX inhibitory activity with IC_50_ values of 14.61 *μ*g/mL. This result is consistent with the previous study of Ghansenyuy et al. [48], reporting the potent lipoxygenase inhibitory activity of *β*-sitosterol-*β-D*-glucoside from the leaves of *Alstonia scholaris*.

The general results showed that compound (2) was less effective compared to compound (1) against NO production and 15-LOX activity. In addition, compound (1) was more active than indomethacin, and quercetin was used as a reference compound to inhibit NO production and 15-LOX activity. This observation suggested that compound (1) is the most promising compound with potential anti-inflammatory properties. Therefore, compound (1) was selected to investigate its effects on COX and inflammatory cytokine release. *D. psilurus* has traditionally been used extensively to treat inflammatory conditions such as rheumatism [9]. In the present study, psoralen was identified as the main active ingredient of *D. psilurus*. Psoralen has also been investigated for its other biological activities, including its effects on immune function, antioxidant, antifungal, and anticancer properties [44].

### 3.4. Effect of Psoralen on Cyclooxygenase Enzyme Activity

**Figure 4:**
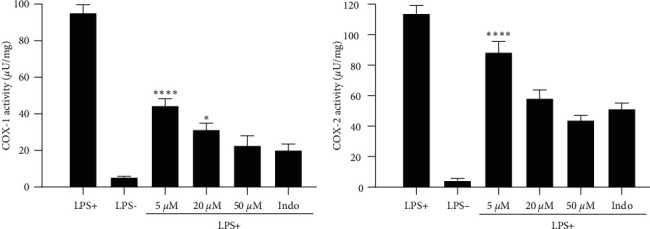
The effect of psoralen on the activity of COX-1 and COX-2 in RAW 264.7 cells stimulated with LPS was investigated. The measurement of COX activity is defined as the production of 1.0 *μ*mol of resorufin per minute by 1 unit (U) of the enzyme under conditions of pH 8.0 and 25°C. Indomethacin (Indo) was examined at a concentration of 10 *μ*M. The reported values represent the average of an experiment carried out in triplicate (*n* = 3) with standard deviation. Statistical analysis was carried out using Dunnett's multiple comparisons test and two-way analysis of variance. The significance levels were denoted as  ^*∗*^*p*  < 0.01 and  ^*∗*^ ^*∗*^ ^*∗*^ ^*∗*^*p*  < 0.0001, indicating the differences in psoralen concentrations compared to the reference drug (indomethacin).

Cyclooxygenases (COX) are integral to the process of inflammation and are responsible for the synthesis of prostaglandins (PGs) [49]. Suppression of COX can lead to a decrease in PG concentration, consequently generating an anti-inflammatory response [50]. In this particular investigation, we explore the impact of psoralen on the enzymatic activity of COX in LPS-induced RAW 264.7 macrophages. The results presented in Figure 4 illustrated that psoralen exhibited concentration-dependent inhibitory effects on both COX-1 and COX-2 activities compared to the untreated control (95.19 and 113.81 *µ*U/mg, respectively). At 5 *μ*M, psoralen showed a more significant inhibitory effect on COX-1 (44.35 *µ*U/mg) than COX-2 (88.33 *µ*U/mg). As the concentration increased to 20 *μ*M, the inhibitory effect was enhanced for both COX-1 (31.25 *µ*U/mg) and COX-2 (58.1 *µ*U/mg). At the highest concentration of 50 *μ*M, psoralen further inhibited COX-1 (22.73 *µ*U/mg) and COX-2 (43.84 *µ*U/mg). Indomethacin, a nonsteroidal anti-inflammatory drug (NSAID) and a COX inhibitor included as a reference drug, inhibited both COX-1 (20.12 *µ*U/mg) and COX-2 (51.29 *µ*U/mg). The anti-inflammatory potential of psoralen, through its targeting of COX enzymes, has been substantiated by previous studies that have focused on psoralen and its derivatives. In a previous study, psoralen isolated from *Dystaenia takeshimana* was reported to show inhibitory effects on both COX and 5-LOX enzymes [51]. Molecular coupling analysis conducted by Ai et al. [52] in 2019 predicted that psoralen possesses COX inhibitory activity and further demonstrated COX inhibitory activity at both mRNA and protein levels in HepG2 cells. Furthermore, a derivative of psoralen named xanthotoxol was found to decrease the protein levels of COX-2 [46]. Our findings confirm that psoralen has the potential to modulate both the COX-1 and COX-2 pathways, thereby positioning it as a promising candidate for the development of a novel anti-inflammatory drug with dual inhibitory effects of COX.

### 3.5. Effect of Psoralen on Cytokine Production

**Figure 5:**
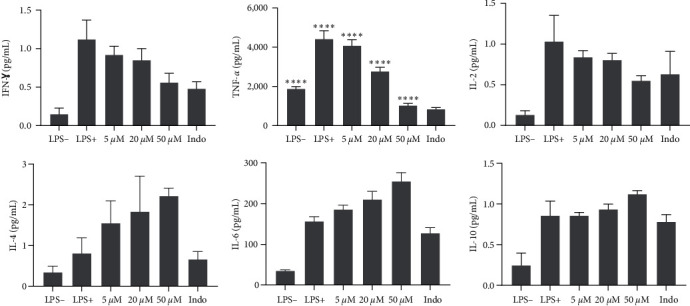
Effects of psoralen (1) and indomethacin on inflammatory cytokines released in LPS-stimulated RAW 264.7 macrophages. Cells were exposed to various concentrations of psoralen for 24 hr, and cytokine levels were quantitatively determined. Indomethacin was tested at a concentration of 10 *μ*M. The results presented here are the mean of one experiment performed in triplicate, with the standard deviation indicated. Statistical analysis was conducted using Dunnett's multiple comparison test with two-way ANOVA. The *p*-values  ^*∗*^ ^*∗*^ ^*∗*^ ^*∗*^*p*  < 0.0001 indicate significant differences between the concentrations of psoralen and the reference drug, indomethacin.

Furthermore, we examine the impact of psoralen on the secretion of Th1/Th2 inflammatory cytokines in LPS-induced RAW 264.7 macrophages. As shown in Figure 5, the compound psoralen exhibited a decrease in the levels of pro-inflammatory Th1 cytokines (IFN-*γ*, TNF-*α*, and IL-2) that depended on its concentration, thus demonstrating its potential as an agent with anti-inflammatory properties. In particular, at a concentration of 50 *μ*M, psoralen significantly reduced IFN-levels of IFN-*γ* (from 1.12 to 0.56 pg/mL), TNF-*α* (from 4,418.86 to 1,032.66 pg/mL) and IL-2 (from 1.032 to 0.551 pg/mL). In comparison, indomethacin, which serves as a reference compound, also showed inhibitory effects on pro-inflammatory cytokines, although to a lesser extent than psoralen. Similarly, psoralen exhibited dose-dependent stimulatory effects on the release of anti-inflammatory cytokines (IL-4, IL-6, IL-10). At the highest concentration tested (50 *μ*M), psoralen significantly improved IL-4 levels (from 0.81 to 2.22 pg/mL), IL-6 (from 156.96 to 255.31 pg/mL), and IL-10 (from 0.857 to 1.122 pg/mL). The anti-inflammatory properties of psoralen, which involve the modulation of inflammatory cytokines, are consistent with previous investigations [53, 54]. The molecule coupling assay revealed that psoralen negatively regulated the expression of inflammatory cytokines (TNF-*α*, IL-1*β*, IL-6, and IL-8) stimulated by *Porphyromonas gingivalis* LPS [55]. Our findings suggest that psoralen may regulate the immune response by suppressing the release of crucial pro-inflammatory mediators. Furthermore, its impact on anti-inflammatory cytokines implies that psoralen can not only inhibit pro-inflammatory responses but also promote anti-inflammatory responses, indicating a dual modulatory effect on immune balance.

Although the anti-inflammatory effects of psoralen are well-documented, it is noteworthy that this study uncovers novel mechanisms of action for psoralen by demonstrating its potent inhibition of 15-lipoxygenase and nitric oxide production in LPS-stimulated RAW 264.7 macrophages. These findings provide new mechanistic insights that deepen our understanding of how psoralen exerts its anti-inflammatory effects. Previous studies have not extensively explored these specific inhibitory effects, making our findings a significant contribution to understanding psoralen's anti-inflammatory mechanisms. Moreover, our work extends the knowledge of psoralen's effects on the immune system by detailing its impact on Th1/Th2 cytokine modulation. This aspect of immune response regulation is critical for developing treatments for a variety of inflammatory and autoimmune diseases. Overall, the comprehensive anti-inflammatory profile presented in our study suggests broader therapeutic applications for psoralen that have not been previously considered. This expands the potential clinical uses of psoralen beyond what is currently known.

As a limitation, this study lacks information on the mechanism underlying its inhibitory effects on psoralen cyclooxygenase enzymes and the inflammatory cytokines studied. Although the results are promising, more studies, including in vivo experiments, exploration of signaling pathways such as NF-*κ*B and MAPKs, and synergistic effects of psoralen with other isolated compounds, are needed to confirm the efficacy and safety of psoralen as an anti-inflammatory agent. It should be noted that in addition to its medicinal properties. *D. psilurus* is widely used in Cameroon as a culinary spice [56]. Although the plant is not considered a rare or endangered species, there are still important environmental and ethical considerations to take into account during its extraction and use [57, 58]. Therefore, the extraction and use of *D. psilurus* and similar plant species should be approached with a holistic perspective that considers environmental sustainability, ethical sourcing, cultural respect, and community involvement [59]. In addition, responsible environmental and ethical practices are essential to ensure the long-term sustainability of both species and ecosystems in which they reside.

## 4. Conclusions

Bioguided fractionation led to the identification of compound 1 (psoralen) as the main anti-inflammatory bioactive compound in the methanol extract of the roots of *D. psilurus*. The isolated and identified psoralen exhibits an anti-inflammatory activity that is dependent on its ability to regulate the production of NO, 15-LOX, and COX-1/COX/2 activities and the release of cytokines in LPS-induced RAW 264.7. These data contributed to the growing body of evidence supporting the anti-inflammatory potential of psoralen and the use of natural compounds in the development of novel anti-inflammatory therapeutics. Further studies, including potential clinical studies, exploration of synergistic effects with other compounds, or investigations and other biological activities, are necessary to understand the underlying mechanisms and potential therapeutic applications.

## Figures and Tables

**Table 1:** IC_50_ values (in *μ*g/mL) of selected extracts, fractions, and compounds isolated from *D. psilurus* in 15-LOX and inhibition of NO in RAW 264.7 macrophage cells.

Samples	NO inhibition	15-LOX inhibition
DP	103.32 ± 8.65^a^	108.54 ± 8.32^a^
DPA	112.45 ± 10.04^a,b^	107.43 ± 9.54^a,b^
DPB	>200	185.32 ± 10.84^c^
DPC	174.67 ± 9.34^c^	178.98 ± 11.87^c,d^
DPA2	101.68 ± 11.14^a,b,c^	99.15 ± 9.08^a,b,e^
DPA3	87.81 ± 7.92^a,c,d^	67.85 ± 5.76^f^
DPC3	180.08 ± 14.65^c,e^	144.28 ± 12.45^g^
DPC4	>200	193.45 ± 8.64^c,d,h^
(1)	1.19 ± 0.45^f^	3.43 ± 0.75^i^
(2)	39.41 ± 2.29^g^	35.57 ± 2.35^j^
(3)	26.15 ± 3.01^h^	14.61 ± 1.38^k^
Quercetin	nd	6.78 ± 0.95^l^
Indomethacin	3.79 ± 0.88^i^	nd

nd, not determined; DP, crude extract; DPA, petroleum ether subextract; DPC, ethyl acetate subextract; DPA1 and DPA3 are fractions of DPA; DPC3 is a fraction of DPC. The significance of the differences between the IC_50_ values was determined using Fisher's least significant difference (LSD) at a level of significance of 5%. Values with different letters are significantly different at *p* < 0.05.

## Data Availability

The data used to support the findings of this study are available upon reasonable request from the corresponding author.

## Supplementary Materials

Supplementary Materials Supplementary material (S1–S12) provides ^1^H, ^13^C, COSY, HSQC, and HMBC NMR spectra for each of the three isolated compounds. S1–S6 for psoralen (1), S7–S11 for stearyl ferulate (octadecanyl-3[4-hydroxyphenyl]-prop-2-enoate) (2), and S12–S16 for *β*-sitosterol glucoside (3).
