# Supplementary material for: Psoralen Isolated from the Roots of Dorstenia psilurus Welw. Modulate Th1/Th2 Cytokines and Inflammatory Enzymes in LPS-Stimulated RAW 264.7 Macrophages
Source: Mediators Inflamm. 2024 Jul 11;2024:8233689. doi: 10.1155/2024/8233689 (PMC11257762; doi:10.1155/2024/8233689)
Supplement: Supplementary Materials — Supplementary material (S1–S12) provides 1H, 13C, COSY, HSQC, and HMBC NMR spectra for each of the three isolated compounds. S1–S6 for psoralen (1), S7–S11 for stearyl ferulate (octadecanyl-3[4-hydroxyphenyl]-prop-2-enoate) (2), and S12–S16 for β-sitosterol glucoside (3). [file 8233689.f1.docx]

**Supplementary Materials**

| **Table of Contents** | | **Page** |
| --- | --- | --- |
| S1: | ^1^H NMR for **Psoralen** (500 MHz, Methanol-*d*_4_)…………………... | 2 |
| S2: | ^13^C-NMR for **Psoralen** (125 MHz, Methanol-*d*_4_)…………………... | 2 |
| S3: | DEPT 90 NMR for **Psoralen** (125 MHz, Methanol-*d*_4_)……………. | 3 |
| S4: | COSY NMR for **Psoralen** (500 MHz, Methanol-*d*_4_)………………. | 3 |
| S5: | HSQC NMR for **Psoralen** (500 MHz, Methanol-*d*_4_)………………. | 4 |
| S6: | HMBC NMR for **Psoralen** (500 MHz, Methanol-*d*_4_)……………… | 4 |
| S7: | ^1^H NMR for *Stearyl ferulate* (*Octadecanyl-3[4-hydroxyphenyl]-prop-2-enoate* (400 MHz, Acetone-*d*_6_)…………………………………….. | 5 |
| S8: | ^13^C-NMR for *Stearyl ferulate* (*Octadecanyl-3[4-hydroxyphenyl]-prop-2-enoate* (140 MHz, Acetone-*d*_6_)…………………………………….. | 5 |
| S9: | HSQC NMR for *Stearyl ferulate* (*Octadecanyl-3[4-hydroxyphenyl]-prop-2-enoate* (400 MHz, Acetone-*d*_6_)………………………………. | 6 |
| S10: | HMBC NMR for *Stearyl ferulate* (*Octadecanyl-3[4-hydroxyphenyl]-prop-2-enoate* (400 MHz, Acetone-*d*_6_)………………………………. | 6 |
| S11: | COSY NMR for *Stearyl ferulate* (*Octadecanyl-3[4-hydroxyphenyl]-prop-2-enoate* (400 MHz, Acetone-*d*_6_)……………………………….. | 7 |
| S12: | ^1^H-NMR for *β-sitosterol glucoside* (600 Mz, DMSO-*d*_6_)……………. | 7 |
| S13: | ^13^C-NMR for *β-sitosterol glucoside* (150 Mz, DMSO-*d*_6_)……………. | 8 |
| S14: | HSQC NMR for *β-sitosterol glucoside* (600 Mz, DMSO-*d*_6_)………... | 8 |
| S15: | HMBC NMR for *β-sitosterol glucoside* (600 Mz, DMSO-*d*_6_)……….. | 9 |
| S16: | COSY NMR for *β-sitosterol glucoside* (600 Mz, DMSO-*d*_6_)………… | 9 |

S1: 1H NMR for **Psoralen** (500 MHz, Methanol-*d*_4_)

S2: ^13^C-NMR for **Psoralen** (125 MHz, Methanol-*d*_4_)

S3: DEPT 90 NMR for **Psoralen** (125 MHz, Methanol-*d*_4_)

S4: COSY NMR for **Psoralen** (500 MHz, Methanol-*d*_4_)

S5: HSQC NMR for **Psoralen** (500 MHz, Methanol-*d*_4_)

S6: HMBC NMR for **Psoralen** (500 MHz, Methanol-*d*_4_)

S7: ^1^H NMR for *Stearyl ferulate* (*Octadecanyl-3[4-hydroxyphenyl]-prop-2-enoate* (400 MHz, Acetone-*d*_6_)

S8: ^13^C-NMR for *Stearyl ferulate* (*Octadecanyl-3[4-hydroxyphenyl]-prop-2-enoate* (100 MHz, Acetone-*d*_6_)

S9: HSQC NMR for *Stearyl ferulate* (*Octadecanyl-3[4-hydroxyphenyl]-prop-2-enoate* (400 MHz, Acetone-*d*_6_)

S10: HMBC NMR for *Stearyl ferulate* (*Octadecanyl-3[4-hydroxyphenyl]-prop-2-enoate* (400 MHz, Acetone-*d*_6_)

S11: COSY NMR for *Stearyl ferulate* (*Octadecanyl-3[4-hydroxyphenyl]-prop-2-enoate* (400 MHz, Acetone-*d*_6_)

S12: ^1^H-NMR for *β-sitosterol glucoside* (600 Mz, DMSO-*d_6_*)

S13: ^13^C-NMR for *β-sitosterol glucoside* (150 Mz, DMSO-*d_6_*)

S14: HSQC NMR for *β-sitosterol glucoside* (600 Mz, DMSO-*d_6_*)

S15: HMBC NMR for *β-sitosterol glucoside* (600 Mz, DMSO-*d_6_*)

S16: COSY NMR for *β-sitosterol glucoside* (600 Mz, DMSO-*d_6_*)
